# Editorial

**Published:** 1869-04-01

**Authors:** 


### EDITORIAL.

"There's cheating in all trades but ours," says the old adage. Recent developments, however, would make it appear that we, like some others, have been reposing serenely under the consolations of the maxim, until its applicability has become a matter of tradition rather than of justice. The statements made by Mr. E. H. Sargent, President of the Chicago College of Pharmacy, at the late annual meeting of that body, with reference to the frauds practiced in the drug trade, demand attention from the whole profession who are, perhaps, more immediately interested in the subject than the pharmacists to whom the remarks were directed.

Coming from such a source these statements must be considered authoritative and reliable, and the grievances which they set forth call loudly for reform. If the advocates of *protection* are so very anxious for the *protection of the public* as they claim to be, let them direct their efforts in this direction, in which the prospect of attaining their object is more promising. Let them induce the Legislature to pass an act (and provide for its enforcement also) to punish with the utmost severity these indirect murderers, who jeopardize and destroy human life for the sake of money.

The vender of "Benzine whisky" is far less culpable than these; he merely offers the option to others to poison themselves; they poison the innocent and unsuspecting.

In the number of The Journal of February 15, 1868, we were so fortunate as to be able to present to our readers a verbatim report of the experiments of Dr. Jules Lemaire, as presented by him to the Academy of Sciences, Paris. We have been still further fortunate in securing a report of Dr. L.'s conclusions from his observations, which we give entire, in the present number.

Dr. J. Hughes Bennett, of Edinburgh, should be heard on the other side of the subject. In a pamphlet reprinted from the *Edinburgh Medical Journal*, for March, 1868, Dr. B. not only denies the justice of Lemaire's conclusions, but also completely demolishes the cell theory. So far as the atmospheric Germ Theory bears upon the question of the origin and propagation of disease, its solution is certainly of practical importance to medical men, and deserves still further investigation as, notwithstanding the labor expended upon the question, it is *adhuc sub judice*.

